# Deconstructing atypical eye gaze perception in autism spectrum disorder

**DOI:** 10.1038/s41598-017-14919-3

**Published:** 2017-11-08

**Authors:** Peter C. Pantelis, Daniel P. Kennedy

**Affiliations:** Indiana University–Bloomington, Department of Psychological and Brain Sciences, 1101 E., 10th Street, Bloomington, IN 47405 USA

## Abstract

The ability to discern the target of another person’s gaze is critical for social and linguistic development, but functions atypically in autism spectrum disorder (ASD). A multi-pronged approach allowed us to deconstruct this complex ability, to uncover the fundamental bases of this impairment. We analyzed performance on a novel gaze perception task with classical psychophysical metrics (precision and accuracy), principal component analysis (in the analysis of spatial biases), and Bayesian computational modeling (in the analysis of individual subjects’ use of contextual salience cues). Compared to controls, adults with ASD were less precise and less accurate in their judgments of gaze direction. Further, although nearly all controls exhibited a prototypical pattern of spatial bias in their judgments, this spatial prior was severely disrupted among a large subset of ASD participants. By contrast, Bayesian computational modeling revealed that both groups exploited contextual salience cues in their gaze judgments, and that the average strength of this contextual prior was similar for both groups. This comprehensive study revealed that although most ASD participants performed atypically in at least one aspect of gaze perception, the particular aspects disrupted varied idiosyncratically across individuals. Impairment in gaze perception in ASD likely arises via heterogeneous underlying mechanisms.

## Introduction

Impairment in the ability to discern the target of another person’s gaze is one of several documented social difficulties associated with autism spectrum disorder (ASD)^1–3^. Gaze is a critical social cue for dynamically tracking the attention of others, and additionally alerts one to potentially important objects or happenings in the shared environment^4–7^. Further, gaze perception facilitates linguistic development, as tracking the attention of the speaker allows for inference about the referent^8^. Thus, atypical development of this complex social ability^9,10^ can have pervasive and persistent effects on perception and cognition in the context of ASD.

Although perception of eye gaze is often considered as a singular ability, like many complex social abilities it can be broken down into several dissociable and more fundamental processes^11,12^. Disruption to any of these underlying mechanisms could therefore result in atypical gaze perception as it manifests in ASD. This study systematically deconstructs eye gaze perception into four more fundamental constructs (precision, accuracy, spatial bias, and integration with context), to ask what specific aspect (or aspects) of this ability lie at the root of of this impairment, and whether the answer to this question is consistent across affected individuals.

Eye gaze perception is partially modulated by relatively low-level perceptual processes^13^, and the human visual system typically extracts the direction of another person’s eye fixation with remarkable precision^14^. In ASD, disruption to early visual processing could lead to affected individuals simply extracting this social perceptual cue in a noisier manner—i.e. with less *precision*. Alternatively, a participant could be highly precise—i.e., reliably reproduce the same response to any respective stimulus—but inaccurate, with these responses being poorly calibrated with respect to correspondence with the underlying ground truth. Importantly, precision and *accuracy* can potentially be dissociated from one another, but this has only rarely been explicitly examined in the context of gaze perception^15^.

Even if eye fixation cues were perceptually extracted with typical fidelity in ASD, the signal would still be accompanied by some noise, creating conditions of partial uncertainty and ambiguity. In the face of these conditions, people rely on *spatial biases* (or priors) to help infer the mostly likely direction of gaze. Notably, people are biased to judge another’s gaze as being in the direction of one’s self^16,17^), and it has recently been posited that this prior is largely intact in high-functioning adults with ASD^18^.

*Context* also helps to resolve this uncertainty and ambiguity; for example, people take into account how salient the potential targets of gaze are—that is, how *a priori* likely various locations are to be looked at^11^. Broadly speaking, that individuals with ASD would weight cues atypically (in both social and non-social domains) has been the topic of much recent theoretical speculation^19,20^. With respect to salience in particular, at least one study has found that adults with ASD may be susceptible to letting salience cues override gaze cues when attempting to discern the referent of a novel word^21^.

This study comprehensively analyzes performance on a novel gaze perception task with two classical psychophysical metrics (precision and accuracy), principal component analysis (PCA; in the analysis of spatial biases), and Bayesian computational modeling (in the analysis of individual subjects’ use of contextual salience cues). This quantitatively rigorous approach—emblematic of the emergent interdisciplinary field of computational psychiatry^22^—allows us to not only uncover mean group differences (control vs. ASD) where they exist, but also to investigate whether disruption to gaze perception in ASD stems from a consistent group deficit, or if the fundamental mechanism of impairment idiosyncratically varies among individuals. Given the high degree of heterogeneity characteristic of ASD, we expect the latter, and this approach will further allow us to parse this heterogeneous performance.

## Methods

**Figure 1:**
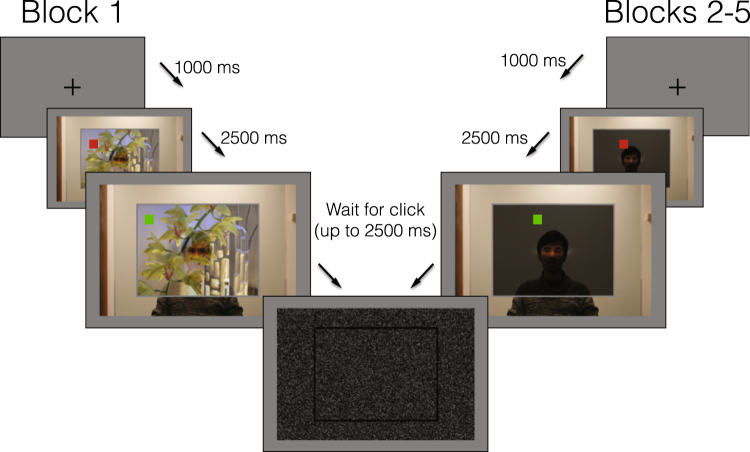
After the presentation of a fixation cross for 1000 ms, the scene appeared, with a mouse cursor appearing as a red square at a random location within the projected image (this image was a photograph in block 1, and uniform gray in blocks 2–5). After 2500 ms, the cursor turned green and the participant indicated with a mouse click where he or she thought the gazer was looking. (*Note:* The fixation crosses and red mouse cursors are enlarged in this figure to be more visible. The projected photograph originates from the LabelMe dataset^42^, made available to the research community without restrictions).

27 adults with ASD and 31 approximately age-, gender-, and IQ-matched controls (see Supplementary Table 1S for full descriptions of the samples) viewed photographs of a person gazing at various target locations on a partially transparent surface, situated between the “gazer” and the camera. Participants judged where on this continuous 2-dimensional surface the gazer was looking (Fig. 1). On most trials (Blocks 2–5), this surface was colored a uniform gray, offering no informative context for participants’ judgments. From these trials, we analyzed the precision, accuracy, and spatial biases exhibited by each participant’s judgments. During Block 1, this surface contained an arbitrary photographic image, allowing us to examine the extent to which individual participants used contextual salience cues to inform their judgments.

This experiment employed stimuli used in a previous study^11^. Unabridged methodological details appear in Supplemental Material. The study was performed in accordance with the tenets of the Declaration of Helsinki, and the experimental methods were approved by the Institutional Review Board of Indiana University. The data generated and analyzed during the current study are available from the corresponding author on reasonable request.

### Participants

Participants were recruited from the Bloomington, Indiana area, and responded to flyers (posted around the community, on the university campus, or on Craigslist) or were referred by word of mouth. All participants gave written informed consent and were compensated at a rate of $15 per hour.

The ASD sample consisted of 27 adults (age 17–55, *M* = 25.1; 22 male) of average or above-average IQ (80–133, *M* = 116.0), all of whom had previously received clinical diagnoses of autism, Asperger’s Syndrome, or Pervasive Developmental Disability-Not Otherwise Specified (PDD-NOS). DSM-IV-TR diagnoses were confirmed using the Autism Diagnostic Observation Schedule-2 (ADOS-2) Module 4—administered by research reliable personnel^23^—and a review of background information and neuropsychological assessments (including IQ testing, Autism Spectrum Quotient [AQ], Beck Depression Inventory, State-Trait Anxiety Inventory, and self-reported clinical history), together with clinical judgment. The suggested ADOS cutoff score is 7; however, in light of empirical evidence that the ADOS Module 4 has very similar specificities at thresholds of 6 and 7 when administered to high-functioning adults^24^, we included participants with an ADOS score of 6 if all other available information supported an ASD diagnosis (n = 3).

The control sample consisted of 31 approximately age- (19–42, *M* = 25.7), gender- (25 male), and IQ- (99–153, *M* = 117.0) matched participants. One additional control was excluded from the final sample because of technical problems with the experiment; another was excluded after participation because he later reported a past diagnosis of schizophrenia.

### Stimuli

**Figure 2:**
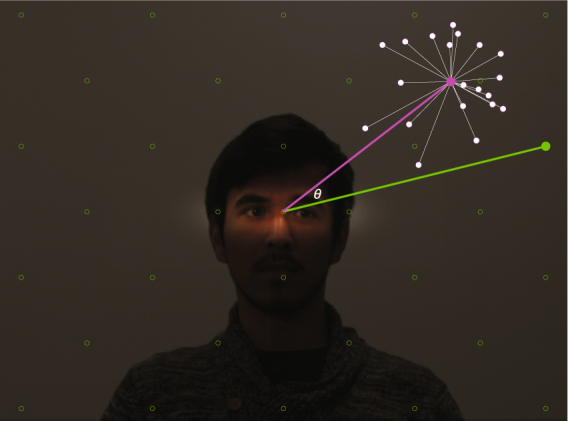
The possible targets of gaze are represented here with green circles; the target of gaze corresponding to this specific photograph is shown with the filled-in green circle. Each stimulus was shown to each participant 20 times; the 20 judgments this individual participant made in response to this stimulus are shown with white circles. The distance of these responses to their mean (white lines connected to the magenta circle) enters into the calculation of the participant’s *precision*. The angle between the mean response to this stimulus (magenta line) and the ground truth target (green line) enters into the calculation of the participant’s *accuracy*.

Photographs were taken of a young man seated behind (and gazing upon) a glass surface, which was situated between him and the camera. One of these photographs was taken with the gazer fixating on the origin (i.e. straight ahead, and directly into the camera), and the other 32 photographs were taken with the gazer fixating on 32 respective locations arranged in a lattice of 7 rows and 9 columns (see green dots in Fig. 2). The gazer maintained minimal head and body movement across these 33 photographs.

All fixated locations fell within a rectangular frame superimposed on presented scenes. Either an image (Block 1) or uniform gray (Blocks 2–5) was presented within the frame in each scene, and alpha blended with the background photograph of the gazer. This created a perceptual effect akin to the participant and gazer being on opposite sides of a semitransparent surface, with the gazer’s silhouette faintly visible through it. Only a tight ellipse around the gazer’s eyes was fully visible through the surface, with the area around the eyes smoothly transitioning to greater opacity. Thus, in all conditions the gazer’s eyes were fully visible to the participant, and presented simultaneously with the supposed target of gaze.

The 165 color images used in Block 1 conveyed a variety of indoor and outdoor scenes, and were selected from a larger, publicly available set^25^.

### Procedure

The experiment was programmed in MATLAB using the Psychophysics Toolbox^26^. It consisted of 5 blocks, each consisting of 165 trials. Over the course of each block, each of the 33 photographs of the gazer (fixating on the 33 respective locations) was featured 5 times, with these 165 total trials being randomly ordered. For Blocks 2–5, the frame in front of the gazer was filled with a uniform gray. For Block 1, one of 165 color images was randomly assigned to each of these 165 trials and projected into the frame; thus, the projected images and the actual target of the gaze were randomly paired. 2.5 s after the onset of the stimulus, the color of the cursor changed from red to green, indicating when the participant was permitted to respond. The participant clicked where he or she believed that the gazer was looking. The scene remained on the screen until the participant responded, or for 2.5 s more (whichever came first). The scene was then replaced with a Gaussian noise mask, and the participant pressed the spacebar to move on.

### Analyses

#### Precision and Accuracy

Precision is defined as how consistently (or inversely, how noisily) a participant responds when presented with the same stimulus over repeated trials. Precision was calculated as the average (squared) error with respect to the overall mean of judgments made by the participant, divided by the average (squared) error with respect to the mean of the condition (i.e. the mean of all responses to a respective gaze pose; see Fig. 2). This yielded a single summary measure of precision for each participant. In Supplementary Figure 1S we also display average (and group differences in) precision with respect to every location in the gazed-upon space.

Accuracy is defined as deviation from a ground truth reference. On a trial-by-trial basis, precision will limit accuracy. But an individual can be accurate and imprecise, to the extent that the mean of his or her repeated responses to the same stimulus tends to converge toward ground truth.

In other words, calculating accuracy on a trial-to-trial basis does not satisfactorily decouple precision from accuracy. We therefore first averaged over the individual’s 20 responses to each single gaze pose (e.g., the magenta dot in Fig. 2), and then calculated the accuracy of this response compared to the ground truth target of gaze (e.g., the green dot in Fig. 2). To the extent that this average persistently deviated from ground truth, it reflected inaccuracy (or bias), and not random noise. We further note that whereas the precision of responses (as we have measured it) could also be potentially influenced by individual differences in visuomotor noise, this factor is likely to cancel out after averaging over repeated trials (i.e., as in our calculation of response accuracy).

**Figure 3:**
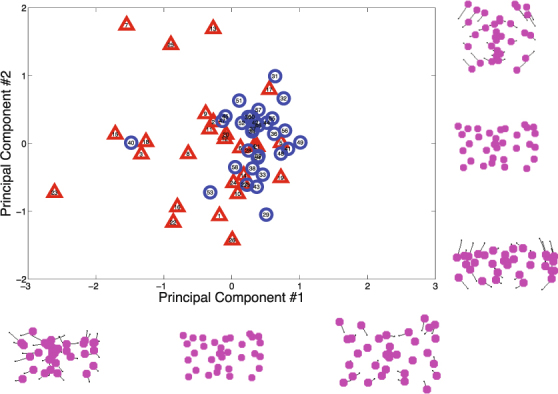
A visualization of the first two components of a PCA of participants’ gaze judgments, with each participant plotted within this reduced-dimensionality space. Participants with ASD are represented with red triangles; controls are represented with blue circles.

We further observed that some participants’ responses were much more spread out than others, overall. This was likely related to how near the participant assumed the gazer was to the gazed-upon surface; this depth dimension was difficult to perceptually infer from the stimuli we used. The closer the participant assumed the gazer was to the surface, the more clustered the responses toward the center, and nearly all participants tended to produce judgments that were much more crowded toward the center of the space than the actual targets of gaze (see Fig. 3 and Supplementary Figure 2S). Further, this tendency appears to have been a trivial individual difference, rather than a meaningful measure of performance, and did not significantly vary by group (operationalized specifically with respect to the mean distance of the subject’s responses from the center of the space; *t*[56] = 0.86, *p* = 0.39). For these reasons, calculating accuracy with respect to discrepancy from ground truth in 2-D space was unlikely to be a useful performance metric, and we determined that angular accuracy—calculated as the cosine similarity between the response and the ground truth target of gaze (e.g., the cosine of *θ* in Fig. 2)—was a more meaningful measure of performance than spatial accuracy.

#### Spatial Bias

Although the ground truth targets of gaze were distributed approximately uniformly over the gazed-upon 2-D surface, participants’ judgments were not. Errors with respect to ground truth showed systematic, non-random biases. A bias toward the vertical center line was typically observed (see also^15^), perhaps resulting from the consistent cue of the gazer’s head being oriented straight ahead in each photo, or perhaps from some version of the “looking at you” effect^17^. A typical participant’s responses tended, overall, to conform to a symmetrical “butterfly” pattern (see Fig. 3 and Supplementary Fig. 2S).

We performed a principal component analysis (PCA) to quantify the extent to which individual participants conformed to similar spatial biases (The details of how input features were selected for the PCA and how participants’ responses were preprocessed have been excerpted to Supplementary Material). If individuals with ASD showed lower accuracy, this approach would further allow us to ask whether all of the individuals with ASD tended to be inaccurate in a similar way, or whether the patterns of individuals’ errors varied idiosyncratically. Using PCA, we placed each individual in a reduced dimensionality space (defined by the first 4 principal components, which explained 63.8% of the variance), in which the similarities and differences among the different participants’ spatial biases could be visualized and quantified.

As a metric of the idiosyncrasy of an individual participants’ biases (i.e. the extent that the participants’ response pattern was not like the others), we calculated the *leverage* of each participant in the reduced space. Leverage, by analogy from linear regression, is a measure of how much influence an individual point has on the configuration of the space, and was recently shown to be an effective means for labeling outliers in PCA^27^.

#### Use of Salience Cues

With minor adjustments, our analysis of participants’ use of contextual salience cues is adopted from an earlier study^11^, which used Bayesian computational modeling to determine the extent to which each individual participant took this information into account when making gaze judgments. For extensive details of this modeling approach, the reader is directed to Supplemental Material.

Salience was computed by each of two methods^25,28^, and served as a proxy for the participant’s reasonable expectation—implicit or explicit—of which locations in a scene would be more or less likely to draw the gazer’s visual attention. The visual salience map for each image was treated as the prior in our Bayesian model for participants’ gaze judgments. The extent to which each participant used the salience prior was estimated by fitting a parameter (*δ*), expressing how much the prior should be weighted to optimize the Bayesian model’s fit to his or her data.

## Results

### Precision and Accuracy

Control participants were significantly more precise (*M* = 5.53) than ASD participants (*M* = 3.66; *t*[56] = 3.16, *p* = 0.003). This effect was of similar magnitude toward the center (Cohen’s *d* = 0.90) and in the periphery (Cohen’s *d* = 0.77) of the gazed targets. Controls were also more accurate (${M}_{control}=\mathrm{0.957,}{M}_{ASD}=0.914$; because of left skew in both groups’ distributions, we used a non-parametric permutation test to demonstrate a significant group difference; $p=0.013$).

**Figure 4:**
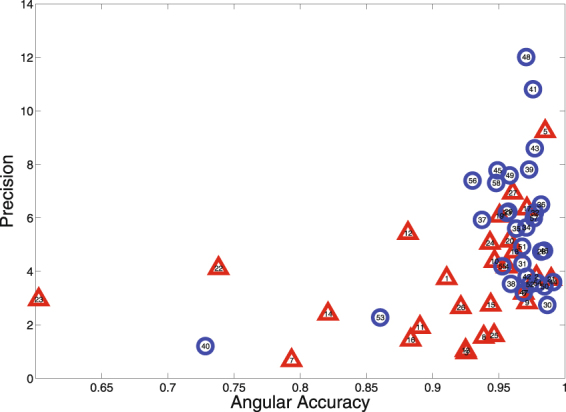
Precision and accuracy of participants’ gaze judgments. Participants with ASD are represented with red triangles; controls are represented with blue circles.

Nearly all control participants and about half of the ASD participants performed in a cluster of high accuracy, with substantial individual differences in precision (see Fig. 4). Though precision and accuracy were correlated (*r*[56] = 0.378, *p* = 0.003), there were notable examples of participants for whom these two measures were not in agreement (e.g., participants #1, 9, 12, and 22).

### Spatial Bias

Figure 3 displays the first two principal components resulting from PCA analysis of participants’ spatial tendencies. The first component (25.0% variance explained) expresses a tendency toward the typical spatial prototype; participants high on this dimension showed strong conformity to the “butterfly” pattern. The second component (19.5% variance explained) represents a tendency to cluster responses toward the horizontal axis (low) versus the vertical axis (high).

**Figure 5:**
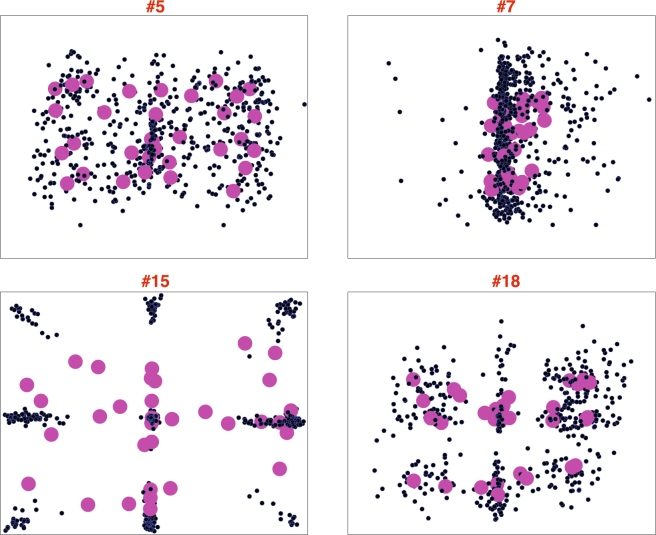
Examples of response patterns produced by four ASD participants. Raw responses are represented with the smaller dots; the large magenta dots represent the mean responses of the participant to each of the 33 respective gaze stimuli. Participant #5 (top left) produced the prototypical “butterfly” pattern. The other three participants shown here produced patterns of responses that not only deviated strongly from the prototype, but from one another.

Almost all control participants and about half of ASD participants were clustered closely in the space, showing a tendency toward the butterfly pattern (Dim. 1), and not much additional bias toward the x-axis or y-axis (Dim. 2). Leverage (i.e. idiosyncrasy in spatial bias) was strongly correlated with accuracy ($r\mathrm{[56]}=-\mathrm{0.737,}\,p < 0.001$), and also showed a weaker (but significant) correlation with precision ($r\mathrm{[56]}=-\mathrm{0.302,}\,p=0.021$). Although half of ASD participants showed typical spatial biases, ASD participants were much more likely to exhibit idiosyncrasy than controls. The distribution of leverage had a heavy tail in the ASD group, and ASD participants on average tended to exhibit higher leverage than controls ($p=0.005$ by permutation test). Figure 5 shows data from 4 example participants from the ASD sample, one of whom conformed strongly to the prototype (#5), and three of whom exhibited drastically different spatial patterns of gaze judgments.

### Use of Salience Cues

**Figure 6:**
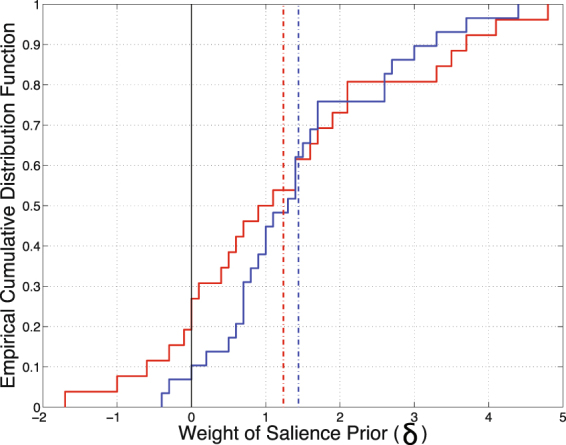
Each of the participants (ASD participants in red, controls in blue) is represented as an increment in these empirical cumulative distribution functions, which illustrate the extent to which the best model fit to each participant’s data exploited a salience map as a prior. Group means are shown with dotted lines.

Both groups weighted contextual salience^25^ significantly above zero (${M}_{control}=1.4$, $\,t\mathrm{[28]}=6.60$, $p < \mathrm{0.001;}\,{M}_{ASD}=\mathrm{1.2,}\,t\mathrm{[26]}=\mathrm{3.88,}\,p < .001$). There was no significant group difference in the use of the salience cue ($t\mathrm{[53]}=\mathrm{0.53,}\,p=0.596$), though the individuals who weighted this cue the most and least tended to be from the ASD sample (Fig. 6). This pattern of results replicated when utilizing an alternative salience algorithm^28^.

Participants’ use of salience was not correlated with any of the other measures (accuracy, precision, spatial bias; all *p* > 0.25).

### Heterogeneity of impairments in ASD

**Figure 7:**
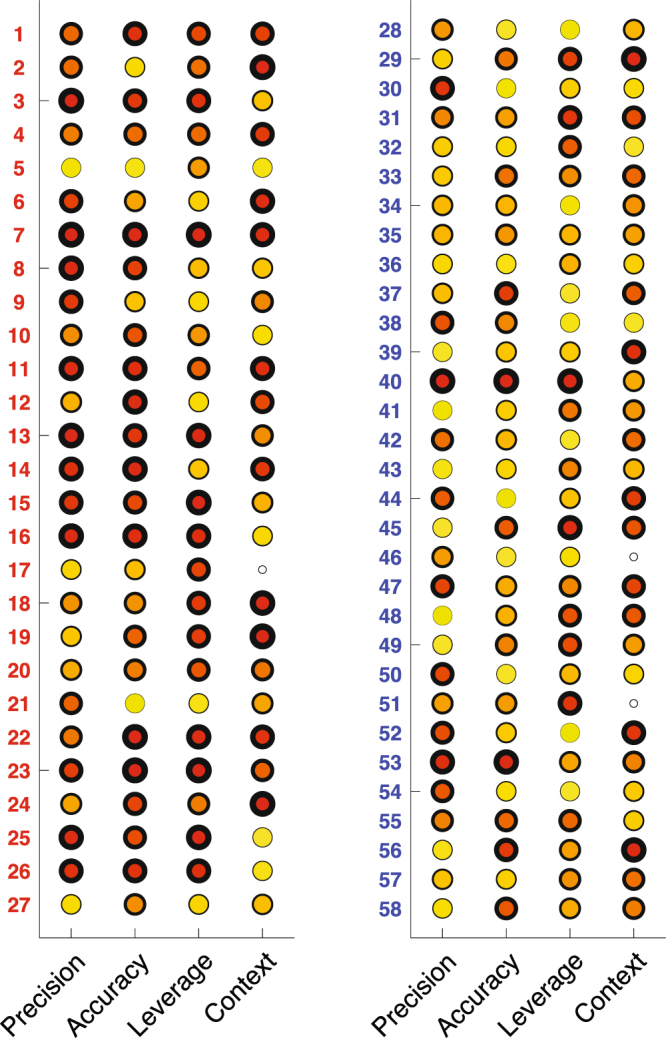
Each of the 27 ASD participants (left) and 31 controls (right) are profiled in this summary figure. Four circles arranged in a row represent respective performance on the four metrics (precision, accuracy, spatial bias [i.e. leverage], and use of contextual salience). The 58 participants in the sample were rank ordered, and their performance percentile along each metric is represented with a circle colored on a gradient from red (with thick outlines, meaning impaired or atypical performance) to yellow (with thin outlines, meaning typical or unimpaired performance). *Note:* 3 participants were excluded from the salience analysis (see Supplementary Material for rationale).

Figure 7 summarizes the performance of every individual in the sample, along each of the four metrics. A few ASD participants showed no discernible impairment on any aspect of the task (e.g. #5 and 27). Likewise, very few were impaired across the board (e.g. #7). Most ASD participants performed atypically in one or more aspects of the task, but not in all of them (e.g. #3, 18, and 19). After ranking all participants across both samples along each of these dimensions, 77.8% of ASD individuals were in the bottom quartile on at least one of the measured dimensions, compared to 29.0% of controls. 33.0% of ASD individuals were in the bottom 5% of the sample on at least one of the measured dimensions; this was only true of one of the controls (#40).

An important aspect of understanding this variability in performance is to examine whether variation along respective metrics reflects meaningful individual differences that relate to other stable traits in individuals with ASD. As an example of this approach, and as an exploratory analysis (especially given the limited sample size), we correlated Autism Spectrum Quotient (AQ) with our four performance metrics. Among our participants with ASD, AQ was not correlated with any of these performance measures (all p > 0.12).

## Discussion

The ability to infer the target of another’s gaze—and the nature of its impairment in autism spectrum disorder (ASD)—has been the focus of a great deal of recent scientific attention^1,13,18,19,29^ and deservedly so: eye gaze perception is fundamental to dynamic social interaction and communication, and critically supports linguistic development. However, as we demonstrate in this paper, gaze perception is not an irreducible social ability, but rather can be unpacked into a variety of more fundamental components. Using an experimental method that relied on the analysis of hundreds of trials from each individual subject, viewed through the lenses of many different performance metrics, we established that performance among control participants tended to cluster within a relatively narrow range. Controls were precise, accurate, exhibited similar spatial biases, and exploited contextual salience cues in the service of their gaze judgments. By contrast, many or most ASD participants performed atypically in one or more aspect of the eye gaze perception task.

However, which particular aspect was disrupted was not necessarily consistent throughout the sample. In other words, idiosyncratic aspects of gaze perception seem to underlie impairments in gaze perception in ASD. Further, a subset of the ASD sample exhibited no discernible impairment at all. We observed nothing systematic that explains this; for example, unlike in an earlier study^1^, we did not find our female participants with ASD—#4, 8, 11, 16, 24—to be specifically spared.

Our approach contrasted with most previous investigations of gaze perception in ASD in several ways. First, our methodology enabled us to successfully dissociate accuracy from precision. In doing so, we discovered that for a subset of participants, impairment on this task was well characterized as arising from noisy (i.e. imprecise) processing of the eye gaze cue. For an overlapping subset of participants, even after averaging over 20 noisy responses to each repeated stimulus, the judgment of gaze never converged toward accuracy with respect to ground truth. In other words, the mapping of the eye gaze stimulus to the perceived direction of gaze was biased, or miscalibrated, in these individuals.

Second, our stimuli were sampled exhaustively from the space of possible gaze directions. This allowed us to uncover fascinating and highly idiosyncratic spatial patterns in the data that would not have been easily discernible in data derived from tasks with less thorough spatial sampling, or those that use adaptive procedures that are typically geared toward the efficient estimation of discrimination thresholds along a given perceptual parameter (e.g.^1^).

Our study was motivated in part to test theories that biases or priors might be attenuated in ASD. As in a previous study^18^, we observed a tendency for individuals from both groups to judge the lateral direction of gaze as being in the direction of the camera. This tendency was captured by Dimension 2 of our PCA solution, which did not systematically differ between the ASD and control samples. That said, spatial biases were plainly idiosyncratic for a subset of the ASD sample (Figs 3 and 5). This same previous study^18^ speculated that individuals with ASD might exhibit biases that develop differently compared to controls; indeed, we provide evidence here that for certain adults with ASD, these spatial priors may diverge quite drastically from the prototype. Whether this bias occurs at the perceptual or decisional level was not resolvable with these data, though it has been previously demonstrated that eye movements themselves tend to differ in this population during gaze discrimination tasks^30^.

A related theoretical debate surrounds the hypothesis that in ASD, the processing of incoming sensory information is less noisy, leading to relatively less reliance on priors^31^. In the analysis of the precision of participants’ gaze judgments, we found contrary evidence. On average, participants with ASD made judgments that reflected diminished precision, suggesting noisier extraction of the social cue. However, it is also possible that group differences in visuomotor precision when indicating a response with the mouse could have contributed to this increased response noise. Unfortunately, we do not have the necessary data to test between these possibilities directly, but this is something that could easily be included in future studies (e.g., with probe trials where participants are simply asked to click a specified location on the screen).

Via computational modeling, we also estimated the extent to which each individual participant used contextual salience information present in the visual scene to infer the direction of gaze. Though salience was a critical cue for the task, there was no overall group difference in its use. There was, however, somewhat greater variability in the ASD sample in the extent to which individuals weighed this cue. Further research—perhaps with a still larger ASD sample—might be necessary to uncover whether a true subset of the ASD group consistently weighs this cue more (or less) than is typical. For an even more sensitive analysis of the nature of this cue integration, it would also be quite useful to derive subject-specific salience maps for each individual. Evidence suggests that individuals with ASD might interpret the relative salience of locations in the environment somewhat differently^32,33^, and therefore could have subtly different expectations (implicitly or explicitly) about where other people are likely to look, indirectly influencing the resultant inference of gaze direction. Alternately, it is possible that certain individuals with ASD interpret salience typically, but weight this salience context more or less than is typical.

Such an approach could better discern the extent to which these individuals find different locations in a scene salient, and to what extent they weigh this contextual cue differently in the service of gaze perception. More broadly, follow-up experimentation like this can help to answer a deeper question: When one finds performance to be variable among individuals with ASD with respect to dissociable aspects of a social task, to what extent is this variation meaningful? In the example of our gaze perception study, does how the individual performs with respect to one of our four constructs meaningfully correlate with analogous aspects of other social and non-social tasks? After all, there are many social and non-social tasks that rely on the accurate and precise processing of a cue, its context, and the integration thereof, in a manner that reveals prior biases. And do relationships exist with other traits or behavioral phenotypes? We argue that uncovering these correlations will allow for great strides in parsing the heterogeneity characteristic of this disorder.

In other words, whether any of the results observed in this study is specific to gaze perception, or reverses in the context of other social and non-social domains, is an empirical question that should be addressed in future experiments. Especially in light of this limitation, we also note that these results do not provide evidence for a causal role of gaze perception in social difficulties of individuals with ASD. Rather than gaze perception difficulties causing impairment, they could also arise as a consequence of reduced and atypical social or other perceptual experiences. Resolving cause and effect would likely require younger participants and longitudinal experimental designs.

That performance in a social task would be heterogeneous in ASD is consistent with previous findings with this population at the behavioral, neural, and genetic levels^34–38^. As we have demonstrated, even when one limits analysis to one specific social task—gaze perception—there are many potential paths to the impairment that manifests in ASD. We analyzed several dissociable aspects of gaze perception, but in naturalistic contexts there remain even other social cues—like head and body position^16,39,40^ or facial expression^41^–that demonstrably inform the judgment of where another person is looking. This study did not directly investigate the processing of these secondary social cues (we instead isolated eye direction as the primary social cue, while intentionally keeping all other constant), but each additional cue that comes to bear on gaze perception presents yet another possible route to impairment.

In sum, we have deconstructed the task of gaze perception into a number of underlying component processes, and have shown that these can be heterogeneously affected across different individuals sharing a diagnosis of ASD. Disruption to any of these processes can account for atypical gaze processing. Overlaid onto this within-task heterogeneity is the fact that social impairment in ASD can result from disruption to any number of social abilities, including those distinct from gaze perception. Those individuals with ASD who showed no discernible impairment to any aspect of our gaze discrimination task no doubt have difficulty with some other social faculty—hence, their diagnosis. The diversity of underlying abilities that contribute to social behavior—any of which may be disrupted in an individual—creates another layer of possible routes to the global social impairment evident in these individuals’ everyday lives. The extent to which individuals with ASD may be “subtyped” in the service of more precise scientific description or clinical intervention will likely depend on the success of research aimed at deconstructing complex social faculties into corresponding components, and connecting the dots^12^.

## Electronic supplementary material

Supplementary Material
