# Supplementary material for: Deconstructing atypical eye gaze perception in autism spectrum disorder
Source: Sci Rep. 2017 Nov 8;7:14990. doi: 10.1038/s41598-017-14919-3 (PMC5678184; doi:10.1038/s41598-017-14919-3)
Supplement: Supplementary file 1 — Supplementary Material [file 41598_2017_14919_MOESM1_ESM.pdf]

Deconstructing atypical eye gaze perception in autism spectrum disorder

Peter C. Pantelis, Daniel P. Kennedy

Department of Psychological and Brain Sciences
Indiana University-Bloomington
1101 E. 10th Street, Bloomington, IN 47405

Unabridged Methodological Details, Supplementary Tables & Figures

Fifty-four of 58 participants were asked upon debriefing how confident they were (on a scale from 1–10) in their ability to figure out where other people are looking in everyday life, relative to the average person. Controls, on average, reported significantly higher confidence in this ability ($M = 7.9$, $SD = 1.0$) than participants with ASD ($M = 5.6$, $SD = 1.9$; $t[52] = 5.48$, $p < .001$; Cohen's $d = 1.52$); in other words, the average individual with ASD thought he or she was about average, whereas the average control thought he or she was well above average (referred to in many other contexts as the above-average or "Lake Wobegon" effect; Alicke et al., 2005).

Stimuli: Photographs of the "gazer"

We took a set of photographs of a person (the "gazer") seated behind a glass surface. In each photograph, the gazer fixated his eyes on a different location on the glass surface, where a grid of points had been marked (later, these marks were digitally removed from the photographs, leaving no observable trace). Though other cues (such as head position) can also be exploited to infer the target of gaze, for this experiment we aimed only to vary the eye cues among these photographs. Therefore, the gazer maintained minimal head and body movement as he fixated on the various locations on the glass surface.

The height of the origin of this grid of points, the camera lens, and the center point between the gazer's eyes was 125 cm. The glass surface was 115 cm from the gazer's

face, and 160 cm from the camera. The gazer's face was lit from above, both from the left and right, so as to avoid casting heavy shadows on his face. The photographs were taken with a Canon EOS Digital Rebel XT camera, a 50 mm lens, 1/125 s exposure time, and no flash. The original resolution of these photographs was 3456×2304 pixels.

Thirty-three photographs were used in the experiment. One of these photographs was taken with the gazer fixating on the origin (i.e. straight ahead, and directly into the camera), and the other 32 photographs were taken with the gazer fixating on 32 respective marks arranged in a lattice of 7 rows and 9 columns. The 1st, 3rd, 5th, and 7th rows of this lattice each consisted of 5 marks, evenly spaced at 10 cm intervals. The 2nd, 4th, and 6th rows of this lattice each consisted of 4 marks, also evenly spaced at 10 cm intervals, but offset by 5 cm with respect to the odd rows (see the lattice of green dots in Fig. 2 of the main document).

The experiment was presented on a 27 in., 2560×1440 pixel display. One of the 33 photographs of the gazer appeared in every trial of the experiment, within a 1200×800 pixel window at the center of the display. The unused, background portion of the display (falling outside of the edges of the 1200×800 pixel window) was made gray.

For every trial, a rectangular gray frame (inner dimensions: 550×733 pixels; outer dimensions: 570×753 pixels) was superimposed on the photograph. When the gazer had been photographed, he had always fixated on locations that would have fallen within this gray frame. Either an image (for block 1) or uniform gray (for blocks 2-5) was presented within the rectangular gray frame in each presented scene, and alpha blended (at alpha = 180, where 0 is fully transparent and 255 is fully opaque) with the background

photograph of the gazer (see Fig. 1 of the main document). For the participant, this created a perceptual effect akin to the participant and gazer being on opposite sides of a partially transparent surface, with the gazer's silhouette faintly visible through it. Only a tight ellipse around the gazer's eyes was fully visible through the image, with the area around the eyes smoothly transitioning to greater opacity. Thus, in either condition (projected image, or uniform gray), the gazer's eyes were made fully visible to the participant, and presented simultaneously with the supposed target of his gaze.

Stimuli: Projected images

During the first block of trials, images were projected onto the plane upon which the gazer had fixated. The 165 color images (a subset of a pool of images provided by Judd, Ehinger, Durand, & Torralba, 2009) included a wide range of indoor and outdoor scenes, 51 of which contained people. We selected this subset of 165 images from the larger pool on the basis that they were all of a consistent size (768 × 1024 pixels). For this experiment, these images were resized to fit the presented 550×733 frame.

Procedure

The experiment was programmed in MATLAB using the psychophysics toolbox (Brainard, 1997; Pelli, 1997). It consisted of 5 blocks, each consisting of 165 trials. Participants were permitted to take short breaks as needed after the completion of a block. Two ASD participants reported fatigue halfway through the experiment, and were therefore permitted to complete the experiment at a later date.

Before the first trial of each block, four photographs were displayed in succession,

each for 1 s. In these four photographs, the gazer was fixated on four respective locations (marked with 8 × 8 pixel black squares) near the four respective corners of the gazed-upon glass surface. This was a "calibration" of sorts for the participant, who could get a sense of how the gazer's eyes were positioned when he had been photographed fixating on the extremes of the glass surface.

Each trial began with a black fixation cross, presented at the center of the screen for 1 s against a gray background. The participant was then presented with a static scene. Over the course of each block of scenes, each of the 33 photographs of the gazer (fixated on 33 respective locations) was featured 5 times, with these 165 total trials being randomly ordered.

For the first block, one of 165 color images (from the Judd et al., 2009 set) was randomly assigned to each of these 165 trials and projected into the frame in front of the gazer; thus, the projected images and the photographs of the gazer were randomly paired, and the contents of the respective images varied independently of the actual target of gaze. Though the scenes were perceptually realistic, the participant was not explicitly instructed that the gazer was (or was not) truly gazing upon an actual physical image present in front of him when the photographs had been taken. Upon debriefing, most participants expressed skepticism that the gazer was actually looking at the photographs, especially after having viewed multiple trials in which the gazer was seemingly fixating on irrelevant areas of the images.

For the 2nd–5th blocks, the frame in front of the gazer was filled with a uniform gray.

At the onset of stimulus presentation, a 8×8 pixel red square appeared at a random location within the frame, and could be controlled with the mouse. After 2.5 s, the color of the red cursor changed to green, indicating to the participant that he or she was now permitted to respond. The participant clicked where, within the frame, he or she believed that the gazer was looking. The scene remained on the screen until the participant responded, or for 2.5 s more (whichever came first). The scene was then replaced with a Gaussian noise mask. Between trials, only a black frame remained visible to the participant, demarcating the edges of where the projected image had been situated. The participant pressed the spacebar to move onto the next trial. This experimental procedure for each trial is illustrated in Figure 1 of the main document.

Experimental trials had an enforced minimum and maximum reaction time (2.5–5 s), to minimize individual differences in speed-accuracy tradeoffs. Indeed, this procedure produced the intended result, in that none of the reported performance measures were significantly correlated with the individual's mean reaction time ($p > .10$ for all correlations). We also found no significant difference in RT between the two groups ($M_{control} = 3.25$ s, $M_{ASD} = 3.21$ s; $t(56) = 0.47$, $p = .64$, Cohen's $d = 0.13$). Thus, group mean differences in any of the observed performance measures are unlikely to be attributable to one group's participants systematically responding more quickly to the stimuli.

Analyses: Precision

Precision is defined as how consistently (or inversely, how noisily) a participant responds when presented with the same stimulus over repeated trials. Another way to frame the analysis of precision is to observe the distributions of responses provided in response to the various stimuli (i.e. the 33 different poses of the gazer), and to ask how distinguishable these distributions are from one another.

We observed that some participants' responses were much more spread out than others, overall. This was likely related to how close the participant assumed the gazer was to the gazed-upon surface; this depth dimension was difficult to perceptually infer from the stimuli we used. The closer the participant assumed the gazer was to the surface, the more clustered the responses toward the center, but this assumption appears to have been a trivial individual difference, rather than a meaningful measure of performance. Therefore, to calculate a participant's precision, we adjusted for the overall level of variance in his or her judgments.

Precision was calculated as the average (squared) error with respect to the overall mean of judgments made by the partic- ipant (i.e. the origin, if the participant is without bias), divided by the average (squared) error with respect to the mean of the condition (i.e. the mean of all responses to a respective gaze pose; e.g., the magenta dot in Fig. 2 of the main document). This is analogous to calculating the F-statistic for ANOVA or model fitting, and is a measure of how much better one can fit the data with knowledge of what stimulus the participant was looking at, compared to relying only on the overall mean response provided by the participant.

By this measure, a participant could be highly precise—i.e., reliably reproduce the

same response to any respective stimulus—without these responses necessarily having any correspondence with the underlying ground truth. Precision is a measure of noise in the response, which is one potential pitfall in gaze perception performance. Across individuals, it is an empirical question whether precision will be correlated with accuracy, and to what extent.

Analyses: Accuracy

Accuracy is defined as deviation from a ground truth reference. On a trial-by-trial basis, precision will limit accuracy. But an individual could be accurate *and* imprecise, to the extent that the mean of his repeated responses to the same stimulus tends to converge toward ground truth. That is, with respect to a given stimulus seen 20 times, the responses may be quite scattered. But if you took the average of these 20 responses, the measurement might align perfectly with ground truth.

In other words, one could calculate accuracy on a trial-to-trial basis, but this would not satisfactorily decouple precision from accuracy. We therefore average over the individual's 20 responses to one single gaze pose (e.g., the green line and dot in Fig. 2 of the main document), and then calculate the accuracy of this response compared to ground truth. To the extent that this average persistently deviates from ground truth, it is a reflection of inaccuracy (or bias) and not just random noise.

Because nearly all participants tended to produce judgments that were more crowded toward the center of the space than the actual targets of gaze (see Fig. 2S), and because there were individual differences in the extent of this tendency, we determined

that angular accuracy was a more meaningful measure of performance than spatial accuracy. More specifically, we calculated accuracy as the cosine similarity between the response and the ground truth target of gaze (e.g., the cosine of θ in Fig. 2 of the main document). For some participants who were inaccurate on a trial-by-trial basis, angular accuracy did converge to ground truth after 20 repeated trials (e.g., participants #8, 15, 18, and 25 in Fig. 3S). Others remained fundamentally and persistently inaccurate (e.g., participants #14, 22, 23, and 40). This confirmed that the constructs of precision and accuracy were, to some extent, dissociable in this sample.

Nearly all control participants and about half of the ASD participants performed in a cluster of high accuracy, with substantial individual differences in precision (see Figure 3 in main document).

Analyses: Principal Components Analysis

We performed a principal components analysis (PCA) to examine the extent to which individual participants conformed to similar spatial biases. In preprocessing participants' responses before PCA, we first noted that there were individual differ- ences in participants' "calibration" to these stimuli. Most participants showed some minor left-right or up-down bias, but this appears to have been a trivial and random individual difference. Thus, to achieve a more meaningful measure of the shape and magnitude of participants' spatial biases, we first centered each participant's responses with respect to his or her own median response (instead of the true origin).

Further, some participants' responses were much more spread out than others,

overall. This was likely related to how close the participant assumed the gazer was to the surface (the closer the participant assumed the gazer was to the surface, the more clustered the responses; see *Analyses: Precision*). This depth dimension was difficult to infer from the stimuli we used, and did not appear to be a meaningful measure of performance. Thus we transformed x- and y-values into $cos(\theta)$ and $sin(\theta)$ (i.e., x and y, normalized by distance from the center). For each participant, we calculated the mean response to each of the 33 gaze poses, and extracted the $cos(\theta)$ and $sin(\theta)$ for that mean location. These 66 values were then entered in as a vector for the principal components analysis.

Analyses: Bayesian Modeling

Our analysis of participants' use of contextual salience cues is adopted from (Pantelis & Kennedy, 2016), which used computational modeling to determine the extent to which each individual participant took this information into account when making gaze judgments. Eye gaze perception is here defined as the inference of the location $[x,y]$ within the continuous 2- dimensional plane where the photographed individual is gazing—$G_{x,y}$—given the gaze directional cue from the eyes of the person—D—and the image presented in that plane—I. Bayes' rule yields the posterior probability distribution, continuous over the 2-dimensional hypothesis space:

$$p(G_{x,y}|D) \propto p(D|G_{x,y})p(G_{x,y})^{\delta}.$$

The *prior*—$p(G_{x,y})$—is equivalent to the relative visual salience of location $[x,y]$ within image I, where salience is a model of where people are *a priori* likely to direct their

visual attention and fixation. Salience was computed by one of two methods: Judd et al. (2009) and Zhang and Sclaroff (2013). For an example of a Judd et al. salience map (corresponding to a photograph of a flower), see Figure 5S. Although the visual salience map is treated as the "prior" in this Bayesian model, as we learned in Pantelis and Kennedy (2016), the processing of this salience cue is not literally "prior" to the stimulus, but actually happens concurrently with the processing of eye cues. Computed salience serves as a simplified proxy (i.e. a model) for a participant's reasonable expectation—implicit or explicit—of which locations in a scene would be more or less likely to draw the gazer's visual attention.

The extent to which the individual uses the salience "prior" is estimated by fitting a parameter (δ) to each participant's data, which expresses how much the salience cue should be weighted to optimize the model (with respect to the cumulative likelihood of the participant's judgments). If δ is set to 0 for an individual participant's best fitting model, then the addition of the salience map did not systematically improve (or hurt) the performance of a model that takes only the eyes of the gazer into account. The higher the δ, the more weight this individual participant apparently assigned to the salience cue. With respect to this salience prior, we make one additional adjustment to salience maps before they enter into the computational model. Because the likelihood function is derived empirically from judgments the individual participant makes about where the gazer is looking within a uniform gray surface, participant's potential spatial biases will already be largely accounted for via the likelihood. Because images tend to be systematically more salient toward the center, using these salience maps without first

correcting for this center bias will result in a computational model that essentially double counts this global tendency. To create salience maps that better reflect local features of individual images (and are, on average, equally salient at any spatial location), we first calculated the average salience map across the set of 165 maps that corresponding to the images in our set. We then divided each of the 165 salience maps by the average salience map, resulting in a set of maps for which no spatial location was systematically more salient than any other location across the set.

Because color is a feature used in both salience algorithms employed in this paper (and many others), these algorithms would have likely been inappropriate models for two participants in the sample who reported some degree of color-blindness (one ASD and one control). We therefore excluded these two participants from this analysis, along with one additional (control) participant for whom there were technical problems with the experiment during Block 1 (participants #17, 46, and 51).

The *likelihood function*—$L(G_{x,y}|D)$—is derived empirically from each participant's gaze judgments recorded during blocks 2–5 (These were the trials for which the gazer was presented as viewing a uniform gray surface). We associate each photograph of the gazer—associated with the gazer's eyes being fixated in 1 of 33 directions—with a 2-D probability map. (Pantelis & Kennedy, 2016) assumed each of these probability maps to be elliptical in shape (i.e. following a bivariate Gaussian distribu- tion). However, such a model proved to be inappropriate for a small number of idiosyncratic participants in this experiment.

For each of the 33 gaze poses, we started with a uniform 2-D distribution over the

768×1024 pixel space, normalized to sum to 1. After collecting responses from each participant as he or she cycled 20 times through the complete set of 33 eye directions, we added 1 to each location selected. We then smoothed the 2-D map with a symmetric 2-D Gaussian kernel ($\sigma = 40$), and renormalized to sum to 1. In this manner we derived each of 33 probabilistic maps, comprising a complete set of personalized likelihood functions. For an example of one elliptical likelihood map derived for one experimental participant with respect to one of 33 directional cues from the gazer's eyes, see Figure 6S. Each probabilistic map represents the model's prediction for the participant's gaze judgment with respect to this particular stimulus, without respect to additional salience cues.

Table 1S: Descriptions of the ASD and control samples. For age, IQ, AQ, and ADOS, group means are provided with standard deviations in parentheses. The ADOS was only administered to the ASD participants. * = Significantly different from ASD group at p < .001.

	**ASD**	**Controls**
N	27	31
**Male/Female**	22/5	25/6
**Age**	25.1 (8.6)	25.7(6.0)
**VIQ**	117.1(14.0)	116.1(12.9)
**PIQ**	110.3(13.0)	114.1(14.2)
**AQ**	32.1(9.0)	17.0(4.7)*
**$ADOS_{comm.}$**	2.9(1.1)	-
**$ADOS_{soc.}$**	6.4(1.4)	-

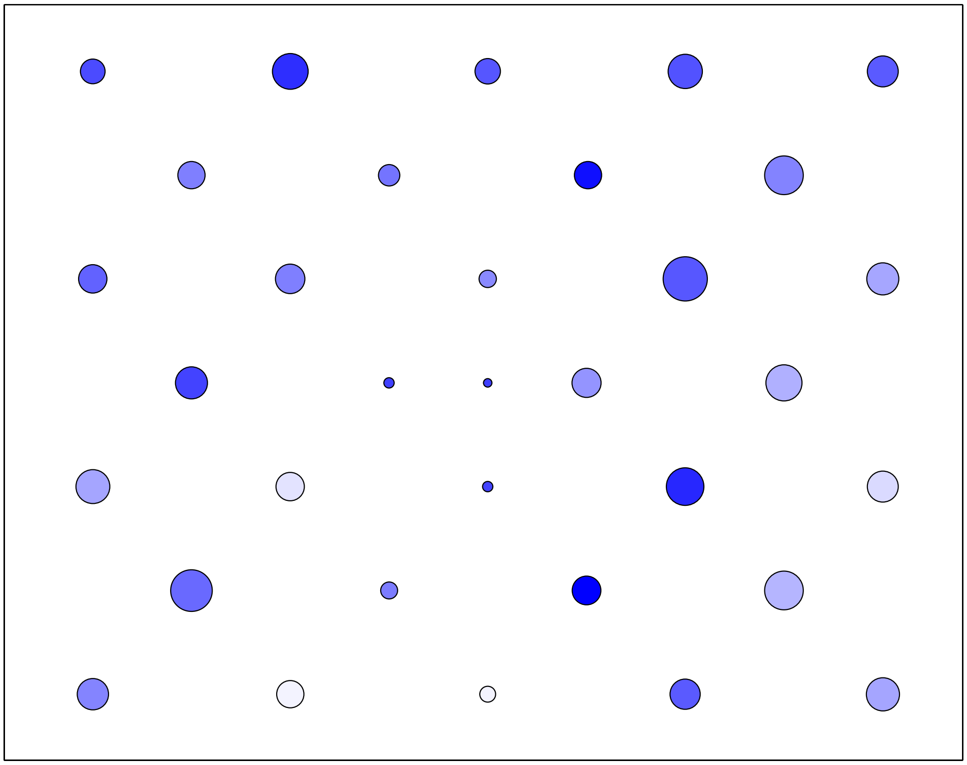

Figure 1S: The precision exhibited by the average subject, with respect to the 33 locations in the gazed-upon space. Precision is in proportion to the size of the circles, with smaller circles representing better precision (e.g., the point in the center resulted in the most precision of response). Larger effect sizes between the control and ASD samples are represented by bluer circles; in every location, controls were (numerically, but not necessarily significantly) more precise. This group-level effect was apparent across the entire space, and therefore not driven by atypicalities in one particular spatial location (e.g., center vs. periphery).

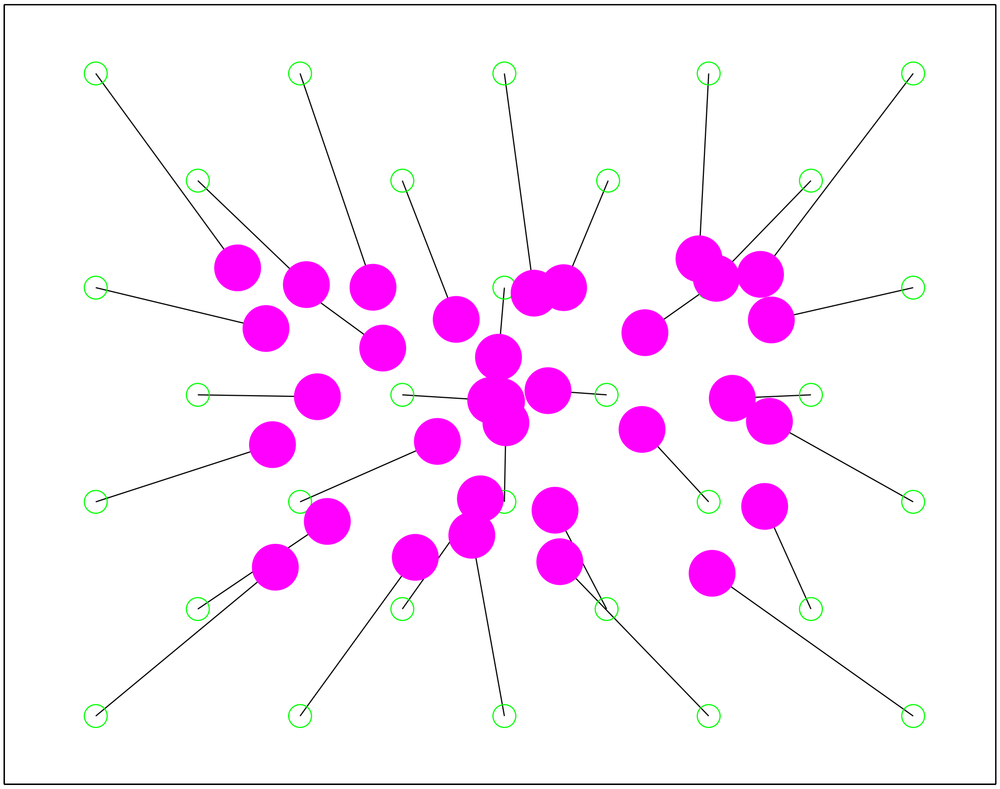

Figure 2S: A representation of the spatial bias exhibited by the average participant. Ground truth targets of gaze are shown with green circles, and connected to the corresponding mean response across participants (filled magenta circles) with a black line.

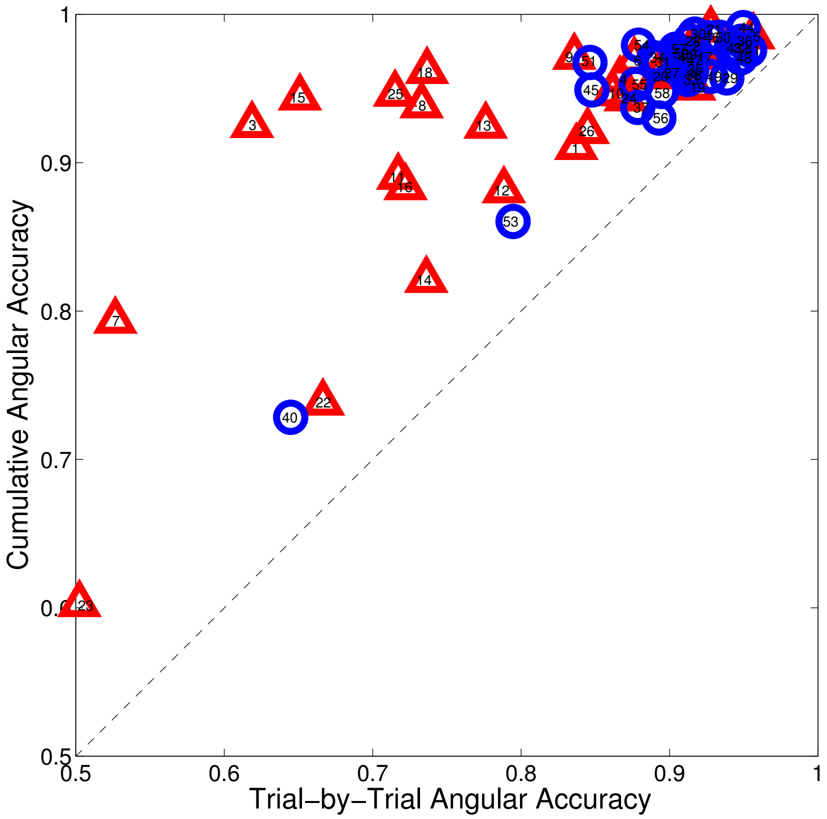

Figure 3S: Accuracy calculated on a trial-by-trial basis (x-axis), compared to accuracy calculated after first averaging over responses to a given stimulus (y-axis). Individual ASD participants are represented with red triangles, and controls with blue circles. The number assigned to each respective participant here is consistently associated with that participant in all subsequent figures, and in the text.

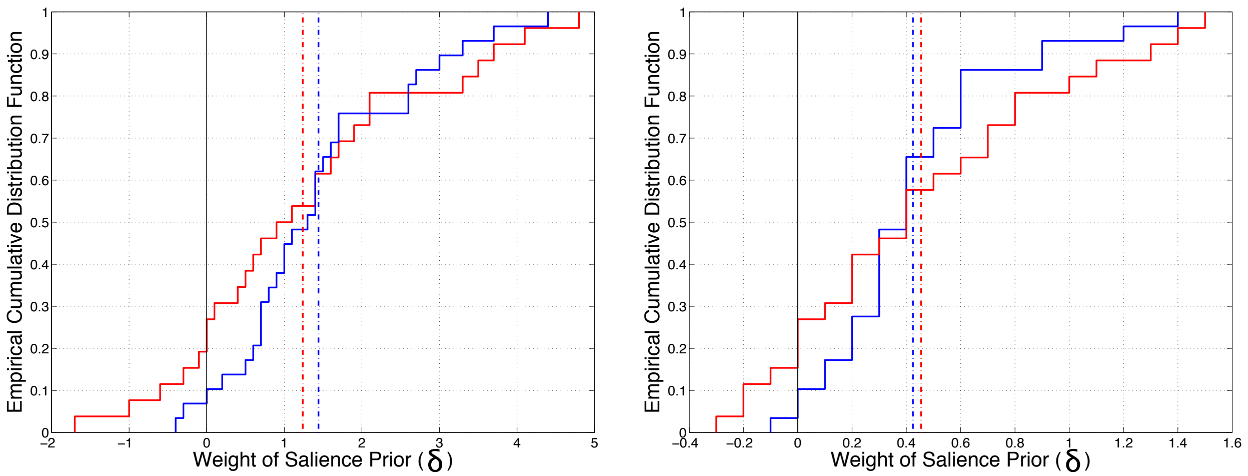

Figure 4S: Each of the participants (ASD participants in red, controls in blue) is represented as an increment in these empirical cumulative distribution functions, which illustrate the extent to which the best model fit to each participant's data exploited a salience map as a prior. In the left panel, this model employed the Judd et al. (2009) algorithm to compute the salience map; in the right panel, the model employed Boolean Map-based Saliency (2013). Group means are shown with dotted lines.

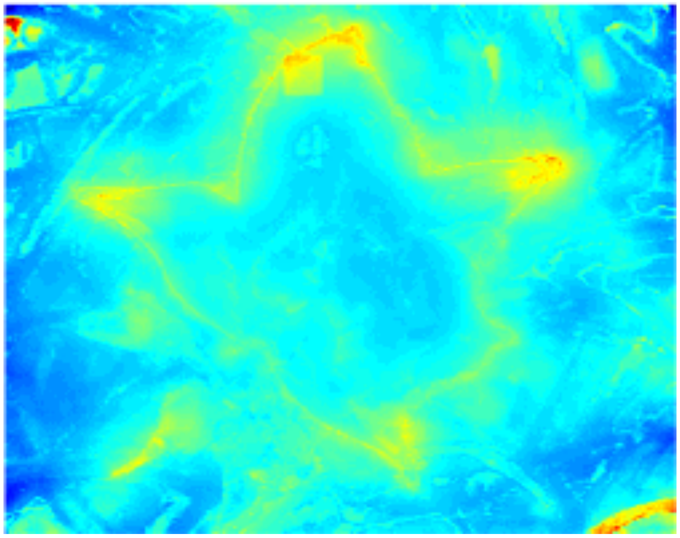

Figure 5S: An example salience map, corresponding to one of the photographs projected onto the gazed-upon surface in Block 1. Here, the gazed-upon image was of a flower.

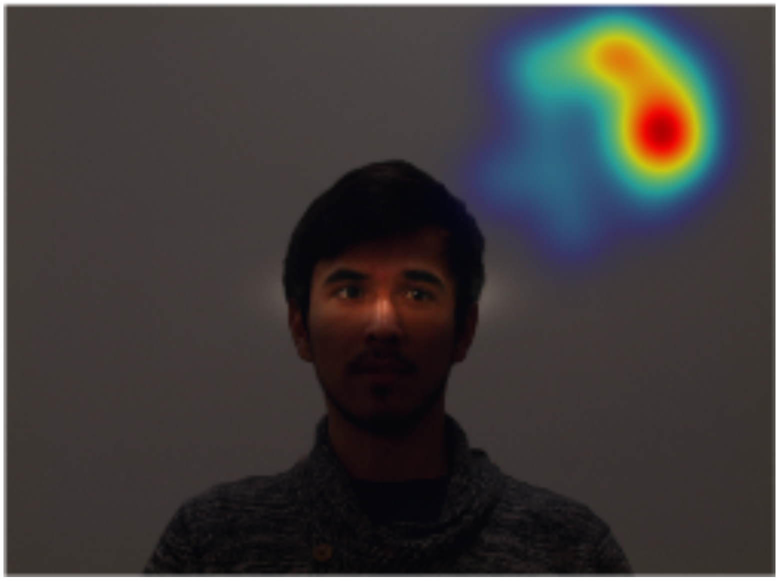

Figure 6S. The participant's 20 responses to this stimulus are smoothed with a Gaussian kernel, to produce the likelihood function (repre- sented here with a heat map) corresponding to when this individual viewed the gazer looking at one target on the surface. The ground truth target of gaze corresponding to this stimuli is shown here with the green dot.

References

Alicke, M. D., Klotz, M. L., Breitenbecher, D. L., Yurak, T. J., & Vredenberg, D. S. (1995). Personal contact, individuation, and the above-average effect. *Journal of Personality and Social Psychology*, *68*, 804–825.

Brainard, D. H. (1997). The psychophysics toolbox. *Spatial Vision*, *10*, 443-446.

Judd, T., Ehinger, K., Durand, F., & Torralba, A. (2009). Learning to predict where humans look. In *IEEE International Conference on Computer Vision (ICCV)*.

Pantelis, P., & Kennedy, D. P. (2016). Prior expectations about where other people are likely to direct their attention systematically influence human gaze perception. *Journal of Vision*, *16*(3:7), 1–12.

Pelli, D. G. (1997). The videotoolbox software for visual psychophysics: Transforming numbers into movies. *Spatial Vision*, *10*, 437-442. Zhang, J., & Sclaroff, S. (2013). Saliency detection: A boolean map approach. *Proceedings of the IEEE International Conference on Computer Vision (ICCV)*.
